# Extended Shortwave Infrared T2SL Detector Based on AlAsSb/GaSb Barrier Optimization

**DOI:** 10.3390/mi16050575

**Published:** 2025-05-14

**Authors:** Jing Yu, Yuegang Fu, Lidan Lu, Weiqiang Chen, Jianzhen Ou, Lianqing Zhu

**Affiliations:** 1 The School of Electro-Optical Engineering, Changchun University of Science and Technology, Changchun 130022, China; 2021200061@mails.cust.edu.cn (J.Y.); fuyg@cust.edu.cn (Y.F.); 2 School of Instrument Science and Opto-Electronics Engineering, Beijing Information Science and Technology University, Beijing 100192, China; lldan_dido@bistu.edu.cn (L.L.); 18363996991@163.com (W.C.); 3 School of Engineering, RMIT University, Melbourne 3000, Australia

**Keywords:** eSWIR, InAs/GaSb/AlSb T2SL, AlAs_0.1_Sb_0.9_/GaSb barrier, bulk detectivity

## Abstract

Extended shortwave infrared (eSWIR) detectors operating at high temperatures are widely utilized in planetary science. A high-performance eSWIR based on pBin InAs/GaSb/AlSb type-II superlattice (T2SL) grown on a GaSb substrate is demonstrated. It achieves the optimization of the device’s optoelectronic performance by adjusting the p-type doping concentration in the AlAs_0.1_Sb_0.9_/GaSb barrier. Experimental and TCAD simulation results demonstrate that both the device’s dark current and responsivity grow as the doping concentration rises. Here, the bulk dark current density and bulk differential resistance area are extracted to calculate the bulk detectivity for evaluating the photoelectric performance of the device. When the barrier concentration is 5 × 10^16^ cm^−3^, the bulk detectivity is 2.1 × 10^11^ cm·Hz^1/2^/W, which is 256% higher than the concentration of 1.5 × 10^18^ cm^−3^. Moreover, at 300 K (−10 mV), the 100% cutoff wavelength of the device is 1.9 μm, the dark current density is 9.48 × 10^−6^ A/cm^2^, and the peak specific detectivity is 7.59 × 10^10^ cm·Hz^1/2^/W (at 1.6 μm). An eSWIR focal plane array (FPA) detector with a 320 × 256 array scale was fabricated for this purpose. It demonstrates a remarkably low blind pixel rate of 0.02% and exhibits an excellent imaging quality at room temperature, indicating its vast potential for applications in infrared imaging.

## 1. Introduction

Compared with traditional shortwave infrared, the extended shortwave infrared (eSWIR) detector (1.7 μm–2.5 μm) has a significant advantage in terms of image quality [1]. In a degraded visual environment (smoke, haze, or specific dust), the eSWIR detector can capture targets at a longer distance [2]. It is widely used in military, earth sciences, satellite remote sensing, and optical communication systems [3]. In addition, the eSWIR spectral imaging capability can also be utilized by NASA for planetary science, primarily to study the Earth’s surface as well as other nearby celestial bodies [4].

To better serve the application fields of eSWIR, a multitude of material systems have been studied. InGaAs based on an InP substrate lattice matching is commonly used for 1.7 μm detection. In order to expand the detection wavelength, the InGaAs needs to increase the In component, which leads to a lattice mismatch. Therefore, the defects of the material will cause serious degradation of the device performance [5]. The HgCdTe material has good wavelength tunability and can cover the eSWIR spectral region. However, the difficulty of growing HgCdTe materials (inhomogeneity and low yield), the high cost of the CdZnTe substrate, and the complex device manufacturing process make this solution insufficient for an inexpensive eSWIR detector [6]. Type-II superlattice (T2SL) materials have the advantages of strong bandgap adjustment flexibility, high carrier effective mass, and good growth uniformity in large areas [7]. InGaAs/GaAsSb T2SL structures can achieve a narrower bandgap than InGaAs or GaAsSb. Its lattice-matching growth on the InP substrate can greatly reduce the dislocation in epitaxy [8]. Sidhu et al. have successfully extended the wavelength of InGaAs/GaAsSb to 2.4 μm [9], and researchers are still trying to expand the wavelength. The M structure based on the InAs/GaSb/AlSb material system has greater carrier effective mass and greater bandgap tuning freedom [10]. A p-i-n homogeneous focal plane array imager based on InAs/GaSb/AlSb has been proved [1].

In order to improve the performance of the device, it is necessary to suppress the dark current density to enhance the specific detectivity and responsivity of the photodetector. The characteristic of nBn architecture is that its unique electronic barrier is designed to block the dark current without hindering the photocurrent [11]. Arash Dehzangi et al. combined the large bandgap electron barrier AlAs_0.1_Sb_0.9_/GaSb with an InAs/GaSb/AlSb nBn structure to realize an eSWIR detector with low, dark current density [12]. The PBn structure can also create a unipolar barrier, which can successfully block the dark current related to the depletion region. It also has the advantage of very low voltage bias dependence. Jiang et al. adopted the PBn structure to suppress the dark current of the device and improve the quantum efficiency [13]. Moreover, the pBin structure can also extend the device’s absorption into the visible light spectrum. It utilizes large bandgap AlAs_0.1_Sb_0.9_/GaSb materials as window layers and designs the band alignment of this heterointerface to effectively extract photogenerated carriers. Arash Dehzangi et al. demonstrated a visible/extended shortwave infrared detector based on the InAs/AlSb/GaSb T2SL [14]. However, the effect of large bandgap AlAs_0.1_Sb_0.9_/GaSb materials on the photoelectric performance of InAs/GaSb/AlSb T2SL eSWIR devices still lacks detailed investigation.

This paper presents an InAs/GaSb/AlSb/GaSb T2SL eSWIR detector based on a pBin structure with a 100% cutoff wavelength of 1.9 μm. By optimizing the doping concentration of the AlAs_0.1_Sb_0.9_/GaSb barrier, the device achieves high-performance specific detectivity. The experimental results are consistent with the simulation results. With the increase in the doping concentration of the barrier structure, the dark current and the responsivity of the device also increase. By separating the bulk dark current and surface dark current of the device, we determined that the optimal doping concentration of the barrier is p-type 5 × 10^16^ cm^−3^. The bulk detectivity of the device is maximized to 2.1 × 10^11^ cm⋅Hz^1/2^/W. The performance of the device at room temperature was improved. A 320 × 256 array-scale eSWIR focal plane array (FPA) detector was further fabricated, and its imaging capability at room temperature was verified.

## 2. Design, Growth, and Fabrication

**Figure 1:**
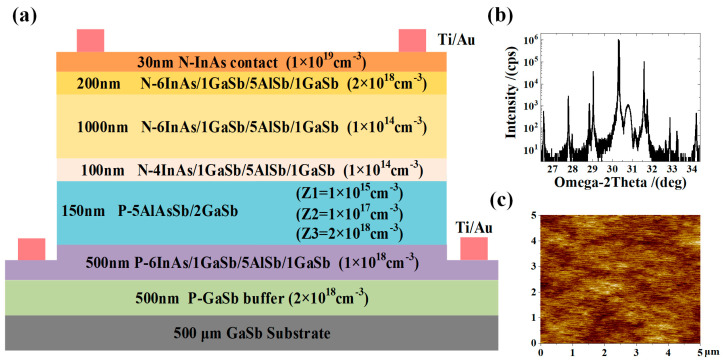
(**a**) Structure diagram of pBin eSWIR photodetector with Ti/Au metal contact layer. (**b**) HRXRD results of sample Z2. (**c**) The RMS roughness of AFM image is 0.125 nm in 5 × 5 μm^2^ area.

The material was grown on an n-type GaSb (001) substrate by the molecular beam epitaxy (MBE) system of Komponenten, Germany. As shown in Figure 1a, it is the designed pBin type eSWIR device structure. Firstly, a 500 nm GaSb buffer layer (p-type 2 × 10^18^ cm^−3^) was epitaxially grown on the GaSb substrate. The bottom contact layer was 500 nm 6/1/5/1 monolayers (MLs) of InAs/GaSb/AlSb/GaSb (p-type 10^18^ cm^−3^). The second electron barrier layer was 150 nm 5/2 MLs of AlAs_0.1_Sb_0.9_/GaSb. Due to the difference in the valence band position between the barrier and the absorption region material, the valence band position of the barrier was adjusted by intentional doping. During the experiment, different beryllium (Be) doping source temperatures were used to achieve precise control of the material doping concentration. Samples doped with Be at varying source temperatures on GaAs substrates were characterized via Hall measurements, enabling precise calibration of the doping concentration corresponding to each Be source temperature. The doping concentrations of Z1, Z2, and Z3 were 1 × 10^15^ cm^−3^, 1 × 10^17^ cm^−3^, and 2 × 10^18^ cm^−3^, respectively. Subsequently, the first electron barrier layer was 100 nm 4/1/5/1 MLs of InAs/GaSb/AlSb/GaSb (undoped, n-type 10^14^ cm^−3^). The absorption region was 1000 nm 6/1/5/1 MLs of InAs/GaSb/AlSb/GaSb (undoped, n-type 10^14^ cm^−3^) T2SL material. The top contact layer was 200 nm 6/1/5/1 MLs of InAs/GaSb/AlSb/GaSb (n-type 2 × 10^18^ cm^−3^), and the last 30 nm InAs (n-type 10^19^ cm^−3^) served as the cap layer of the device. Figure 1b,c are high-resolution X-ray diffraction (HRXRD) and atomic force microscopy (AFM) images of sample Z2, respectively. The HRXRD satellite peak results show that the periodic thicknesses of the absorption region, first barrier region, and second barrier region are approximately 3.85 nm, 3.37 nm, and 2.07 nm, respectively. The stress mismatch between the above superlattice materials and GaSb was within 0.1%, which is consistent with the superlattice design. AFM displays clear atomic steps on the surface of the material, with a Root Mean Square (RMS) roughness of only 0.125 nm in the 5 × 5 μm^2^ region. The surface quality of the material is high. Samples Z1 and Z3 have similar lattice quality and surface quality.

The three devices were prepared using the same batch device-manufacturing process. The circular mesa was wet-etched with a C_6_H_8_O_7_:H_3_PO_4_:H_2_O_2_:H_2_O (5 g:2 mL:1 mL:20 mL) solution. The solution has an etching rate of about 150 nm/min and can precisely control the etching depth to the p-type bottom contact layer. These devices have not been passivated. Finally, Ti/Au (10 nm/100 nm) was deposited on the InAs cap layer (n-type) and bottom contact (p-type) using electron beam evaporation for metallization.

## 3. Results and Discussion

### 3.1. Experiment Result

**Figure 2:**
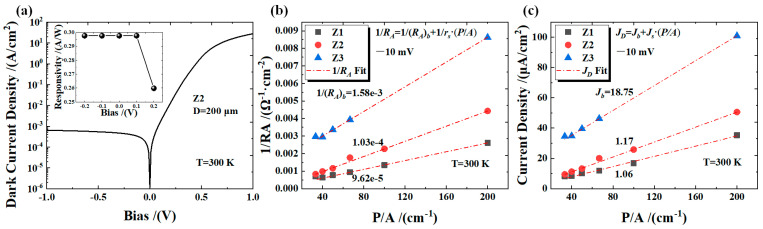
(**a**) The dark current density of device Z2 with a diameter of 200 μm, along with its responsivity under different bias conditions. (**b**) The dark current density for different device sizes is fitted. (**c**) The differential resistance area for different device sizes is fitted.

To verify the electrical performance of different devices at 300 K, the current and voltage characteristics of devices Z1, Z2, and Z3 were measured. The experiment used a semiconductor analyzer (B1500A) as the voltage source and applied a bias voltage to the device through the probe arm of a low-temperature probe station (CRX-VF). The total dark current of the device included bulk dark current and surface dark current. In order to investigate the effect of AlAs_0.1_Sb_0.9_/GaSb barrier on the dark currents of devices Z1, Z2, and Z3, it was necessary to separate the surface dark currents of the devices. Therefore, the current–voltage characteristic curves of devices with different sizes were tested. The devices had radius sizes of 100 μm, 200 μm, 300 μm, 400 μm, 500 μm, and 600 μm, respectively. Figure 2a displays the IV characteristics of device Z2, showing low dark current under a zero/reverse bias and responsivity saturation at −10 mV. The bulk current density (*J_b_*) and surface dark current density (*J_s_*) of the device can be separated by the following equation [15]:

(1) $$J_{D}=J_{b}+J_{s}\cdot (P/A)$$

where *J_D_* is the total dark current density of the device, P is the mesa perimeter, and A is the mesa area of the device. As shown in Figure 2b, the *J_D_* (at −10 mV) of devices Z1, Z2, and Z3 were extracted. The *J_D_* of device Z1 is the smallest, and the *J_D_* of device Z3 is the largest. Subsequently, Equation (1) was used to fit the *J_D_* of different devices. The fitting results show that the *J_b_* of devices Z1, Z2, and Z3 are 1.06 × 10^−6^ A/cm^2^, 1.17 × 10^−6^ A/cm^2^, and 1.875 × 10^−5^ A/cm^2^, respectively. Additionally, *J_D_* is also showing an increasing trend. The bulk differential resistance area (*R_A_*)*_b_* of the device is separated in the same way for the bulk detectivity calculation of the device. The fit uses the following equation [16]:

(2) $$\frac{1}{R_{A}}=\frac{1}{{\left(R_{A}\right)}_{b}}+\frac{1}{r_{s}}\frac{P}{A}$$

where *R_A_* is the total differential resistance area of the device, and *r_s_* is the surface resistivity of the device. The fitting results show that the 1/(*R_A_*)*_b_* of devices Z1, Z2, and Z3 are 9.62 × 10^−5^ Ω^−1^·cm^−2^, 1.03 × 10^−4^ Ω^−1^·cm^−2^, and 1.58 × 10^−3^ Ω^−1^·cm^−2^, respectively. The (*R_A_*)*_b_* of the device shows a decreasing trend as the doping concentration of the AlAs_0.1_Sb_0.9_/GaSb barrier increases.

**Figure 3:**
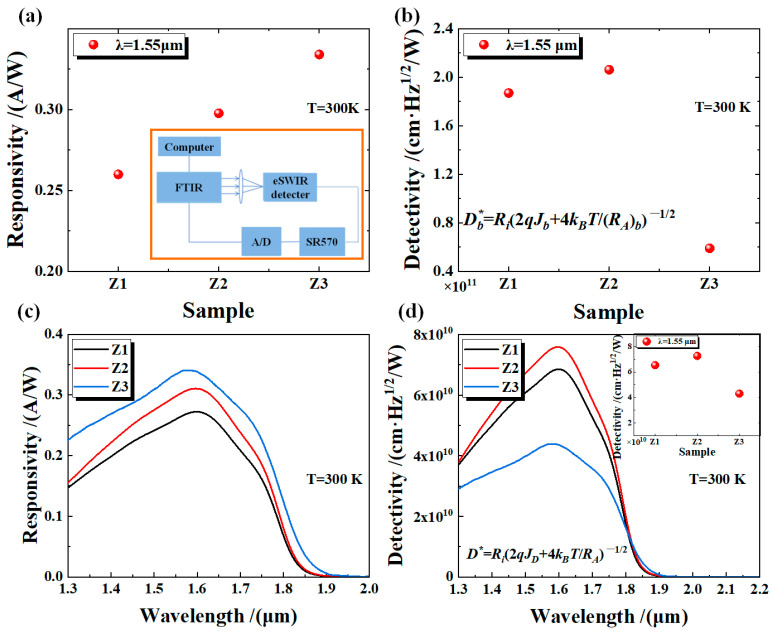
(**a**) The responsivity of devices Z1, Z2, and Z3 under blackbody testing. The lower right corner is schematic diagram of FTIR spectrum response test system. (**b**) The values of the bulk detectivity of the device without *J_s_* at 1.55 μm. (**c**) The normalized responsivity of devices Z1, Z2, and Z3 under a −10 mV bias. (**d**) The spectral specific detectivity of these devices with a diameter of 1200 μm.

To verify whether there are differences in the optical properties of different devices under 300 K conditions, we employed a Fourier-transform infrared (FTIR) spectrometer (Bruker FTIR VERTEX 80/80v, BRUKER, Berlin, Germany) to conduct spectral response tests on devices Z1, Z2, and Z3. The mercury lamp light source was employed to illuminate the detector surface through a CgF_2_ beam splitter. By setting the sensitivity of the current amplifier (SR570) to 200 μA/V, the optical signal received by the device was amplified and then converted into spectral information via an analog-to-digital converter. The background spectrum was acquired using a DTGS detector. The spectral responses of devices Z1, Z2, and Z3 were obtained by subtracting the background spectrum. Finally, the device responsivity was measured using a calibrated 1273K blackbody source. As shown in Figure 3a, the responsivity of devices Z1, Z2, and Z3 at a wavelength of 1.55 μm is 0.26 A/W, 0.30 A/W, and 0.33 A/W, respectively. Furthermore, the spectral response test results show that the 100% cutoff wavelength of all three devices was about 1.9 μm. Then, the spectral response curves were normalized by using the responsivity at 1.55 μm to obtain the responsivity across the full wavelength range (Figure 3c). Therefore, the device responsivity increases with the increase of AlAs_0.1_Sb_0.9_/GaSb doping concentration. This phenomenon shows the same variation trend as the *J_b_* of the device. Although device Z3 has a high responsivity, it also has a large *J_b_*. To further evaluate the comprehensive performance of devices Z1, Z2, and Z3, we neglected the *Js* component of the devices and calculated their bulk detectivity $D_{b}^{*}$ using *J_b_* and (*R_A_*)*_b_*. The calculation formula is as follows:

(3) $$D_{b}^{*}=\frac{R_{i}}{\sqrt{2qJ_{b}+\frac{4k_{B}T}{{\left(R_{A}\right)}_{b}}}}$$

where *R_i_* is the device responsivity, q is the fundamental charge, *k_B_* is the Boltzmann constant, and *T* is the device operating temperature (at 300 K). Figure 3b shows that the $D_{b}^{*}$ of devices Z1, Z2, and Z3 are 1.8 × 10^11^ cm·Hz^1/2^/W, 2.1 × 10^11^ cm·Hz^1/2^/W, and 5.9 × 10^10^ cm·Hz^1/2^/W, respectively. The $D_{b}^{*}$ of the device shows a trend of first increasing and then decreasing. Device Z2 exhibits the best comprehensive performance and has an improvement of 256% compared to device Z3. In addition, we also calculated the specific detectivity $D^{*}=R_{i}{\left(2qJ_{b}+4k_{B}T/R_{A}\right)}^{-1/2}$ [17,18] of devices Z1, Z2, and Z3 (with a diameter of 1200 μm) under a full spectrum. As shown in Figure 3d, device Z2 has the highest specific detectivity (its dark current density is 9.48 × 10^−6^ A/cm² at a bias of −10 mV), and the peak specific detectivity is 7.59 × 10^10^ cm·Hz¹/²/W at 1.6 μm. Device Z2 improved by approximately 73% compared to device Z3. Table 1 shows the performance comparison between device Z2 and other eSWIR detectors. The specific detectivity of our device has certain advantages compared to other detectors. The detector proposed in this work demonstrates notable advantages and exhibits potential for the fabrication of high-temperature eSWIR focal plane array detectors.

### 3.2. Simulation and Discussion

Aiming at the problem that the doping concentration of AlAs_0.1_Sb_0.9_/GaSb changes the current of the device, we used simulation tools to further explain the experimental results. In this study, the performance of semiconductor devices was numerically simulated using Technology Computer Aided Design (TCAD) models [22], including mobility models (parallel electric field-dependent model), recombination models (Shockley–Read–Hall recombination model, Auger recombination model, and optical recombination model), and carrier statistics models (Fermi–Dirac statistics model). The properties of superlattice materials were calculated by the weighted average of InAs, GaSb, AlSb, and AlAs compounds [23]. These included conduction band density (*N_c_*), valence band density (*N_v_*), electron mobility (mun), hole mobility (mup), and dielectric constant. Moreover, the bandgap energy of superlattice materials was calculated using the 8 kp model [24]. Table 2 shows the partial parameter values (at 300 K) of 6/1/5/1 MLs of InAs/GaSb/AlSb/GaSb, 4/1/5/1 MLs of InAs/GaSb/AlSb/GaSb, and 5/2 MLs of AlAs_0.1_Sb_0.9_/GaSb superlattice materials. The model was solved by the Newton and Gummel combinatorial iterative method.

**Figure 4:**
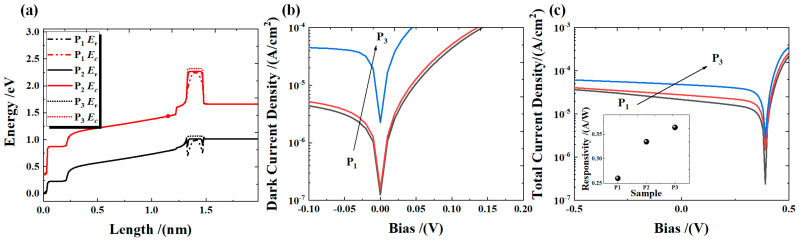
(**a**) The Ec and Ev diagram of the material. (**b**) The variation trends of dark current density under different doping concentrations in the AlAs_0.1_Sb_0.9_/GaSb barrier region. (**c**) The total current density and corresponding responsivity.

We simulated the effects of AlAs_0.1_Sb_0.9_/GaSb with varying doping concentrations on the optoelectronic performance of the devices. The InAs material at the top of the device was taken as the origin, and the opposite direction of material epitaxy was taken as the positive direction to establish the coordinate system. The conduction and valence bands of the material (at −10 mV) are shown in Figure 4a. This includes a 30 nm InAs cap layer (n-type 1 × 10^19^ cm^−3^), a 200 nm top contact layer (n-type 2 × 10^18^ cm^−3^) serving as a photogenerated hole carrier extractor, a 1000 nm absorption region (unintentionally doped, n-type 1 × 10^14^ cm^−3^) for generating photogenerated carriers, a 100 nm first electron barrier region (unintentionally doped, n-type 1 × 10^14^ cm^−3^), a 150 nm second electron barrier region (p-type: P_1_ = 1.5 × 10^16^ cm^−3^, P_2_ = 5 × 10^16^ cm^−3^, P_3_ = 1.5 × 10^18^ cm⁻³), and a 500 nm bottom contact layer (p-type 1 × 10^18^ cm^−3^) acting as a photogenerated electron extractor. The geometric size of the modeled device was 5 × 1 μm^2^.

Figure 4a shows that the first and second electron barriers with a wide bandgap in the device structure can reduce the partial generation–recombination dark current. The second electron barrier can also block the passage of thermally excited electrons [4]. The valence band maximum energy *E_v_* and the conduction band minimum energy *E_c_* both shift upward as the doping concentration of AlAs_0.1_Sb_0.9_/GaSb increases. Figure 4b shows the simulation data of the dark current under different bias voltages. The results indicate that the dark current of the device increases with the rising p-type doping concentration in AlAs_0.1_Sb_0.9_/GaSb. This may be caused by the high hole concentration of AlAs_0.1_Sb_0.9_/GaSb. Under thermal excitation, holes pass through the first electron barrier region to reach the top contact layer, resulting in a larger dark current. Additionally, at the illumination of 1.55 μm, we can simulate the photocurrent data in Figure 4c. Under a −10 mV bias, the photocurrent increases with the increase of AlAs_0.1_Sb_0.9_/GaSb doping concentration. In the absorption region, photogenerated carriers (holes and electrons) are generated under optical excitation, with the top contact layer extracting electrons and the bottom contact layer extracting holes. Due to the upward shift of the *Ev* in the AlAs_0.1_Sb_0.9_/GaSb barrier, the photocurrent increases as the bottom contact layer extracts more photogenerated hole carriers. The simulated dark current densities of devices P1, P2, and P3 are 1.041 × 10^−6^ A/cm^2^, 1.445 × 10^−6^ A/cm^2^, and 1.929 × 10^−5^ A/cm^2^, respectively. These values exhibit close agreement with the bulk dark current density measured experimentally in Figure 2c. Similarly, the responsivity shown in Figure 4c aligns well with the experimental responsivity results from Figure 3a. These observations indicate that there is a difference in doping concentration between Be doped on GaAs and Be doped on AlAsSb/GaSb. Consequently, the actual doping concentration should be determined based on the simulation results. Therefore, the AlAsSb/GaSb doping concentration of Z1, Z2 and Z3 is 1.5 × 10^16^ cm^−3^, 5 × 10^16^ cm^−3^, and 1.5 × 10^18^ cm^−3^, respectively.

### 3.3. Imaging Verification

**Figure 5:**
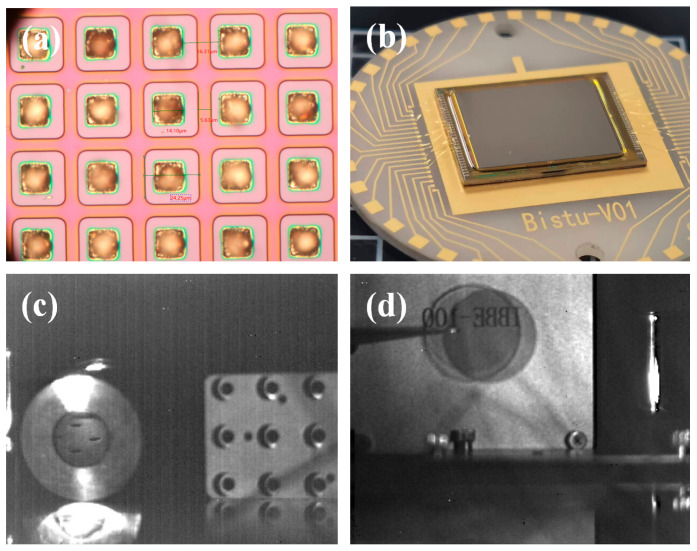
(**a**) Image of the In bump deposition optical microscope; (**b**) eSWIR FPA wiring test diagram; and (**c**,**d**) are eSWIR FPA images at 300 K.

To further validate the imaging capability of the device under room-temperature operation, FPA processing was performed on device Z2. Figure 5a shows the optical microscopy image captured after the indium (In) bump deposition. Negative photoresist lithography was precisely employed to create 12 μm × 12 μm openings, followed by the deposition of In bumps (~7 μm thick, 14 μm × 14 μm in size). Subsequently, the FPA was hybridized with the readout integrated circuit (ROIC) using an FC-150 flip-chip bonder. The gap between the FPA and ROIC was filled to stabilize the GaSb substrate thinning process, achieving a final thickness of ~30 μm. As shown in Figure 5b, the chip was packaged into a dewar for imaging tests. The array scale was 320 × 256 with a pixel pitch of 30 μm. At 300 K and an integration time of 12 ms, the blind pixel rate remained exceptionally low at 0.02%. Figure 5c,d demonstrate sample images captured using an f/2 lens. The FPA successfully resolved the internal structure of an incandescent lamp under intense illumination and achieved silicon wafer-transparent imaging. These results confirm that the eSWIR FPA is a viable candidate for high-temperature, high-performance infrared imagers.

## 4. Conclusions

In summary, an InAs/GaSb/AlSb T2SL photodetector based on the optimization of the AlAs_0.1_Sb_0.9_/GaSb barrier has been designed. Both experimental and simulated studies employed device structures with three different p-type doping concentrations in the AlAs_0.1_Sb_0.9_/GaSb barrier. The results consistently demonstrate that the current increases with elevated p-type doping concentrations in the barrier. Therefore, we evaluated the device performance by comparing bulk detectivity $D_{b}^{*}$. Although the responsivity of device Z3 was improved, the excessively high doping concentration in the AlAsSb/GaSb barrier region caused the junction region to shift toward the absorption region, leading to a sharp rise in dark current. In contrast, device Z2, with an appropriately optimized doping concentration and lower dark current density, achieved a ~73% enhancement in detectivity D* (7.59 × 10^10^ cm·Hz^1/2^/W at 1.6 μm). Additionally, the detector exhibits a 100% cutoff wavelength of 1.9 μm at 300 K, with a dark current density of 9.48 × 10^−6^ A/cm^2^ under a −10 mV bias. A high-performance eSWIR FPA detector with a 320 × 256 array scale was further developed. The test shows that the FPA detector has an effective pixel rate of 99.98% at 300 K, which can accurately capture complex scenes, including resolving the internal structure of incandescent lamps under intense illumination and performing silicon wafer-transparent imaging. These results fully demonstrate its potential for room-temperature eSWIR imaging and detection systems.

## Figures and Tables

**Table 1:** The performance of eSWIR photodetectors is compared with other eSWIR photodetectors.

	Temperature (K)	Cutoff Wavelength (μm)	Specific Detectivity (cm·Hz^1/2^/W)
Ref. [19]	300	1.6	1 × 10^10^ (0 V)
Ref. [20]	293	1.8	6.5 × 10^10^ (-)
Ref. [21]	300	1.8	3 × 10^8^ (0 V)
This paper	300	1.9	7.59 × 10^10^ (−10 mV)

**Table 2:** Some material parameters are used for TCAD model simulation.

Material	Bandgap/eV	*N_c_*/cm^−3^	*N_v_*/cm^−3^	mun/cm^−3^/(V·s)	mup/cm^−3^/(V·s)	Dielectric Constant
6InAs/1GaSb/5AlSb/1GaSb	0.65	1.4 × 10^17^	9.1 × 10^18^	27,717	702	13.4
4InAs/1GaSb/5AlSb/1GaSb	0.75	1.7 × 10^17^	1.1 × 10^19^	20,219	772	12.4
5AlAs_0.1_Sb_0.9_/2GaSb	1.25	8.2 × 10^16^	6.7 × 10^18^	2720	914	15.4

## Data Availability

The data supporting this study’s findings are available within the article.
